# Batwing versus Wise pattern mammoplasty for upper pole breast tumours: a detailed comparison of cosmetic outcome

**DOI:** 10.1186/s12957-017-1124-5

**Published:** 2017-03-14

**Authors:** Tarek Hashem, Ahmed Farahat

**Affiliations:** 1 Breast Surgery Department, National Cancer Institute, Cairo University, Cairo, Egypt; 2 Department of Surgical Oncology, National Cancer Institute, Nr 1, Fom El Khalig, Kasr El Aini Street, Cairo, Egypt

## Abstract

**Background:**

The aim of this study is to compare the results of batwing mammoplasty and Wise pattern mammoplasty in the surgical management of upper pole breast tumours.

**Methods:**

This is a retrospective observational study including 126 breast cancer patients who presented between 2009 and 2015 to the National Cancer Institute of Cairo University in Egypt. All patients were candidates for breast conservation, with upper pole tumours, which was defined as tumours between 3 and 9 o’clock. Patients were categorized into two groups. Group A (64) included patients who underwent inferiorly based therapeutic mammoplasty, while group B (62) was designated for patients who had batwing mammoplasty. The results of both groups were compared and analyzed.

**Results:**

Wise pattern mammoplasty group had statistically significant higher complication rate. The overall aesthetic result of Wise pattern technique was superior to batwing mammoplasty. However, batwing mammoplasty showed a statistically significant higher rate of patient satisfaction.

**Conclusions:**

Both techniques are valid options for upper pole breast tumours. Wise pattern therapeutic mammoplasty remains aesthetically superior; however, batwing mammoplasty is an easy, simple technique with acceptable results to patients.

## Background

Breast-conserving surgery has been well established for the management of early stage breast cancer [1]. The advent of oncoplastic breast surgery has allowed women with breast cancer not only to preserve their breasts but also to retain their aesthetic appearance [2]. Several oncoplastic techniques have been described in order to serve this goal [3]. A number of factors influence the surgeon’s choice of the surgical technique. Tumour size and location, as well as breast size and degree of ptosis, are some of the main factors considered in the decision-making [4].

Upper pole tumours of the breast form a challenge to some extent to the oncoplastic surgeon, especially when considering the aesthetics of the breast. Scars in this area are very unsightly. In addition, less volume of tissue is available in the upper half of the breast to reconstruct the lumpectomy defect [5].

Several oncoplastic techniques have been proposed in order to deal with this problem. Among those techniques are batwing mammoplasty and the inferiorly based Wise pattern therapeutic mammoplasty [6, 7].

Although both techniques are described for the same tumour location, many differences exist between the two approaches. In this study, we try to analyze and compare both techniques, in order to highlight their differences and find out which of them is more suitable to which type of patient.

## Methods

The records were reviewed for all patients who underwent breast-conserving surgery at the National Cancer Institute of Cairo University between 2009 and 2015. Inclusion criteria for the study were as follows:

1. Patients who presented with upper pole breast tumours, which were defined as tumours occurring between and including 3 and 9 o’clock.
2. Patients who underwent either batwing or inferiorly based Wise pattern mammoplasty.
3. Patients who completed their adjuvant treatment and did not lose follow up.
4. Only patients with complete records were included.

One hundred and twenty six patients were identified. They were categorized into two groups. Group A included patients who had inferiorly based Wise pattern mammoplasty (64). Group B was designated for patients who had batwing mammoplasty (62).

Patients were invited to answer a five-scale questionnaire evaluating their own cosmetic outcome graded as excellent (5), good (4), fair (3), poor (2) or very poor (1). Cosmetic criteria they were asked to evaluate were symmetry, shape, volume, projection, correction of ptosis, visibility of the scars and overall satisfaction. Affection of nipple sensation was assessed separately in the same questionnaire.

**Fig. 1:**
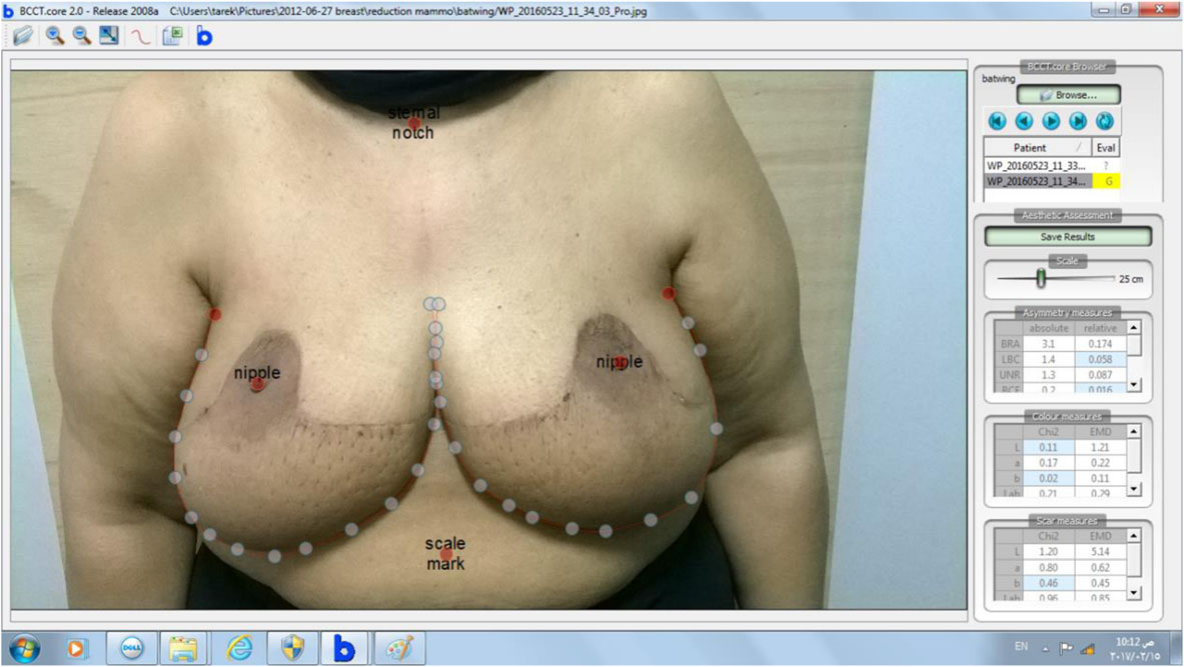
BCCT.core processing a patient’s final picture

Further objective evaluation of the cosmetic outcome was done using the BCCT.core20© software developed by INESC Porto Breast Research Group [8]. The final photograph of each patient was processed by the software and given an overall cosmetic result. The software evaluates antroposterior views of patients. The program provides digital marks for the nipples, suprasternal notch and the axillae. The first step is to allocate these marks to their corresponding anatomical locations on the patients’ photograph. The software then automatically identifies the contour of the breasts (Fig. 1). The result is based on evaluation of three criteria: asymmetry, scar visibility and colour match. Each criterion is assessed through several variables that are automatically calculated. For instance, asymmetry is evaluated through pBRA (the relative breast retraction assessment), the relative difference in nipple position in each breast; pUNR (the relative upward nipple retraction), the relative difference between nipples’ level to each other; pBCE (the relative breast compliance evaluation), the relative difference between the distance of each nipple to inframammary fold; and pBAD (the relative breast area difference), the relative difference between areas of the left and right breasts [8]. Scar visibility and colour match are evaluated in a similar manner. The software automatically calculates and processes all the variables for all criteria and gives an overall cosmetic result.

Data were statistically described in terms of mean ± standard deviation (±SD), and, or frequencies (number of cases) and percentages when appropriate. Comparison of numerical variables between the study groups was done using the Student *t* test for independent samples in normally distributed data and Mann-Whitney *U* test for independent samples in not normal data. For comparing categorical data, chi square (*χ* ^2^) test was performed. Exact test was used instead when the expected frequency is less than 5. *p* values less than 0.05 was considered statistically significant. All statistical calculations were done using computer program SPSS (Statistical Package for the Social Science; SPSS Inc., Chicago, IL, USA) release 15 for Microsoft Windows (2006).

### Patients

The mean age of patients in group A was 44 years with a range of 25–48 years. Group B patients had a mean age of 47 years with a range of 36–58 years.

Mean body mass index (BMI) of group A was 38.2 kg/m^2^ (30.3–41).In group B, the mean BMI was 35.7 kg/m^2^ (28.4–39.3).

Three patients in group A had hypertension and four had type II diabetes. Six patients in group B had type II diabetes, and one patient had rheumatic heart disease.

Preoperative biopsy showed invasive ductal carcinoma in 52 patients and invasive lobular carcinoma in 12 patients in group A. In group B, there were 54 cases with invasive duct carcinoma and 8 cases of invasive lobular carcinoma.

Fifteen patients of group A received preoperative neoadjuvant chemotherapy with the intent of downstaging, while in group B, there were 19 patients.

According to the American Joint Commission on Cancer staging system, patients had the following clinical staging at the initial diagnosis (Table 1):

**Table 1:** Clinical staging of patients at initial diagnosis

Stage	Number of patients
Stage IA	*2*
Stage IIA	*20*/23
Stage IIB	*27*/20
Stage IIIA	*15*/19

Group A (italics); group B (upright)

## Results

### Operative time

Group A had a mean operative time of 220 min with a range of 182 to 254 min, while in group B, the mean operative time was 103 min with a range of 78 to 136 min.

### Hospital stay

Group A had a mean hospital stay of 3 days (range 2–5 days), while group B had a mean hospital stay of 2 days (range 1–3 days).

### Complications

#### Early (less than 2 months)

- Group A: there were two cases of superficial areolar sloughing. Ten cases had wound gapping and delayed wound healing at the T-junction. There was one case of stitch sinus and two cases of axillary seroma.
- Group B: three cases had minor wound infection, and two cases had a breast seroma.

#### Late (more than 2 months)

- Group A: there were two cases of radiation mastitis. Another four patients developed hypertrophic scar. Two other cases had fat necrosis.
- Group B: three cases developed fat necrosis, and other four cases had hypertrophic scars.

### Overall complication rate

The complication rates are shown in Table 2.

**Table 2:** Complication rates

Group A	36% (23)
Group B	19.35% (12)
*p* value	0.038

### Pathological results

- Group A: average tumour size was 3.3 cm, and margins range was 0.7–4.2 cm. Sentinel node biopsy was done in 16 cases. Fourteen patients had a negative sentinel node, and two had a positive sentinel node.
- Group B: average tumour size was 3.6 cm, with a margins range 1.6–5.4 cm. Fifteen patients had sentinel lymph node biopsy. Of these, twelve had a negative node and three had a positive one.

### Cosmetic outcome

The result of each cosmetic criterion, as evaluated by patients, was recorded for each group. The results were then compared and statistically analyzed. Nipple and areola sensory affection and overall satisfaction were also extracted from patients’ questionnaires. The overall cosmetic outcome was finally assessed in each group by evaluating the patients’ end result pictures via BCCT.core20© software [8].

#### Shape

The cosmetic results of shape are shown in Table 3.

**Table 3:** Cosmetic results of shape

Shape	Group A	Group B
Excellent	82.8% (53)	69.35% (43)
Good	12.5% (8)	24.2% (15)
Fair	3.1% (2)	6.45% (4)
Poor	1.6% (1)	
Very poor		
*p* value		0.075

#### Volume

The cosmetic results of volume are shown in Table 4.

**Table 4:** Cosmetic results of volume

Volume	Group A	Group B
Excellent	84.4% (54)	74.2% (46)
Good	9.4% (6)	14.5% (9)
Fair	6.2% (4)	11.3% (7)
Poor		
Very poor		
*p* value	0.360	

#### Ptosis

The cosmetic results of ptosis are shown in Table 5.

**Table 5:** Cosmetic results of ptosis

Ptosis	Group A	Group B
Excellent	89% (57)	83.9% (52)
Good	9.4% (6)	9.7% (6)
Fair	1.6% (1)	6.4% (4)
Poor		
Very poor		
*p* value	0.440	

#### Projection

The cosmetic results of projection are shown in Table 6.

**Table 6:** Cosmetic results of projection

Projection	Group A	Group B
Excellent	76.6% (49)	24.2% (15)
Good	20.3% (13)	17.7% (11)
Fair	3.1% (2)	38.7% (24)
Poor		19.4% (12)
Very poor		
*p* value	˂0.001	

#### Symmetry

The cosmetic results of symmetry are shown in Table 7.

**Table 7:** Cosmetic results of symmetry

Symmetry	Group A	Group B
Excellent	79.7% (51)	16.1% (10)
Good	14% (9)	33.9% (21)
Fair	6.3% (4)	27.4% (17)
Poor		16.1% (10)
Very poor		6.5% (4)
*p* value	˂0.001	

#### Scar visibility

The cosmetic results of scar visibility are shown in Table 8.

**Table 8:** Cosmetic results of scar visibility

Scar visibility	Group A	Group B
Excellent	28.1% (18)	64.5% (40)
Good	45.3% (29)	24.2% (15)
Fair	15.6% (10)	6.5% (4)
Poor	11% (7)	4.8% (3)
Very poor		
*p* value	˂0.001	

#### Overall satisfaction

The overall patients’ satisfaction is shown in Table 9.

**Table 9:** Overall patients’ satisfaction

Overall satisfaction	Group A	Group B
Excellent	40.6% (26)	72.6% (45)
Good	51.6% (33)	6.5% (4)
Fair	4.7% (3)	17.7% (11)
Poor	3.1% (2)	3.2% (2)
Very poor		
*p* value	˂0.001	

#### Sensory affection of nipple and areola

Four patients in group A (6.25%) reported sensory affection of nipple and areola complex. No cases were reported in group B.

#### Cosmetic outcome using BCCT.core20©

The overall cosmetic results evaluated by BCCT.core20© are shown in Table 10.

**Table 10:** Overall cosmetic results evaluated by BCCT.core20©

	Group A	Group B
Excellent	50% (32)	22.6% (14)
Good	29.7% (19)	40.3% (25)
Fair	20.3% (13)	37.1% (23)
Poor		
Very poor		
*p* value	0.005	

## Discussion

In this study, two techniques of volume displacement suitable for upper pole breast tumours were compared. Batwing mammoplasty is a technique that is easy to learn and perform. The procedure is well accepted by patients. The pre-operative markings are easy to perform and could be done according to the patients’ convenience, either in the semi-sitting or standing position.

The results of the study show a statistically significant difference in complication rate in favour of the batwing procedure (Table 2). This is quite plausible for a simple procedure that does not entail complex surgical maneuvers.

The cosmetic outcome was thoroughly investigated in this study in two ways. On the one hand, it was subjectively assessed using patient questionnaires. The questionnaires were structured in a way to allow patients’ evaluation of each cosmetic criterion of their outcome. The aim was to highlight and analyze the different cosmetic aspects of each technique. Thus, questionnaires showed a statistically significant advantage of the Wise pattern technique in projection and symmetry (Tables 6 and 7).

As regards correction of ptosis, shape, and volume, there was no statistically significant difference between both groups (Tables 3, 4 and 5).

On the other hand, the batwing mammoplasty group was significantly more satisfied with scar visibility and overall outcome (Tables 8 and 9).

In order to overcome the inconsistencies of questionnaire derived data, the cosmetic outcome was objectively assessed using the BCCT.core20© software. The program showed a statistically significant superiority of the cosmetic outcome of the Wise pattern group (Table 10).

There are a number of facts to be considered when evaluating the results displayed in this study.

First, this is a retrospective non-randomized study evaluating the outcome in a peculiar group of patients. The numbers assigned to each group were the number of patients who underwent these procedures during the period of the study. Further prospective randomization is needed to validate the results obtained in this study. The majority of Middle Eastern women has a conservative dress code and is less worried about a scar in the cleavage area. Nevertheless, these scars, when carefully placed and sutured tend to heal perfectly and fade out by time.

Second, bilateral Wise pattern mammoplasty was associated with a higher complication rate and affection of nipple and areola sensation in 6.25%. This probably had its impact on overall patients’ satisfaction.

Recent literature records nipple and areola sensory affection to be very rare with inferior pedicle reduction [9, 10]. However, some earlier reports have reported rates as high as 40% [11]. Inferior pedicle reduction definitely carries less risk of sensory affection of nipple and areola when compared to other pedicles; still, there is some risk of occurrence of this complication.

Further correlation between different cosmetic criteria and patient satisfaction is warranted in future studies. Questionnaires assessing patient-reported aesthetic outcomes should identify which cosmetic criteria patients put most value on. Results could help in further innovations of oncoplastic techniques or bring about improvements in skin closure material.

## Conclusions

**Fig. 2:**
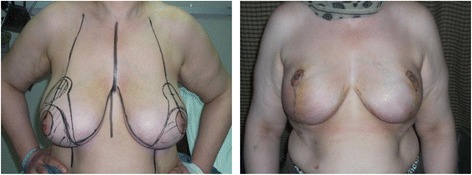
A case of bilateral Wise pattern mammoplasty

**Fig. 3:**
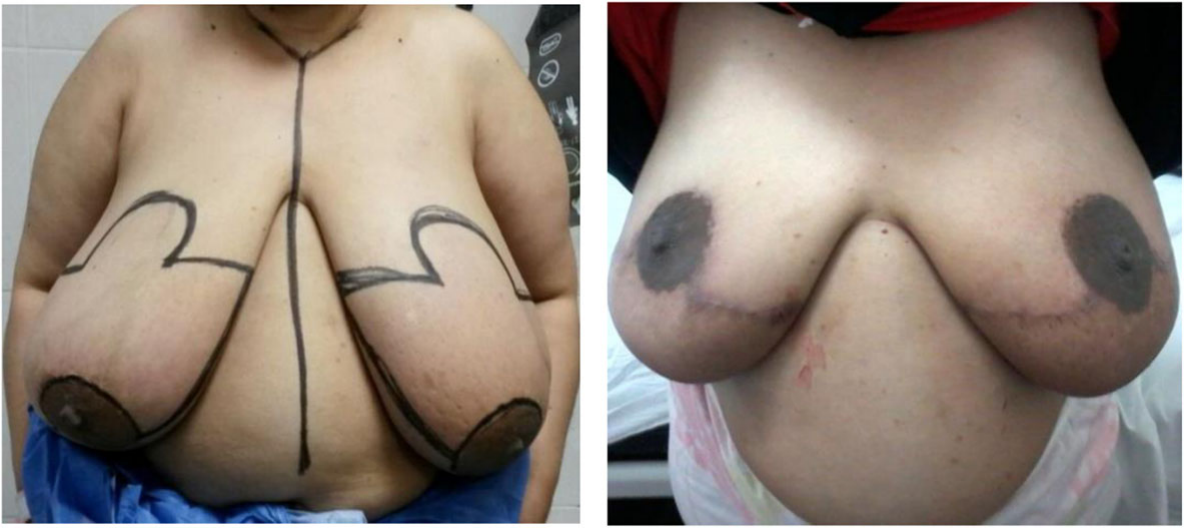
A case of bilateral batwing mammoplasty

In light of the experience displayed in this study, the batwing mammoplasty technique has proven to be a simple and quick procedure for upper pole breast tumours. It will result in an acceptable cosmetic result with a relatively low risk of post-operative complications when compared to Wise pattern therapeutic mammoplasty. The batwing procedure is not only suitable for patients but is very appropriate for surgeons at the beginning of their oncoplastic careers. It is a rather easy to learn surgical technique with very forgiving results. In addition, the suture line of batwing mammoplasty could be easily incorporated within a future mastectomy incision line, in case a mastectomy was indicated along a patient’s course of treatment (Figs. 2 and 3).

On the other hand, Wise pattern therapeutic mammoplasty remains a more cosmetically appealing technique that achieves superior aesthetic outcome. It is associated, however, with more complications and a risk of some degree of sensory loss over nipple and areola. It would be more suitable for younger patients with no significant medical co-morbidities and patients who put significant value on their cosmetic result. There is a significant learning curve required to master this technique. Surgeons who are a bit advanced in their oncoplastic career should perform it.
